# Unveiling the Low-Lying Spin States of [Fe_3_S_4_] Clusters via the Extended Broken-Symmetry Method

**DOI:** 10.3390/molecules29092152

**Published:** 2024-05-06

**Authors:** Shibing Chu, Qiuyu Gao

**Affiliations:** School of Physics and Electronic Engineering, Jiangsu University, Zhenjiang 212013, China; gaoqiuyuuu@gmail.com

**Keywords:** iron–sulfur clusters, density functional theory, Extended Broken-Symmetry method, magnetic coupling constant, low-lying spin state

## Abstract

Photosynthetic water splitting, when synergized with hydrogen production catalyzed by hydrogenases, emerges as a promising avenue for clean and renewable energy. However, theoretical calculations have faced challenges in elucidating the low-lying spin states of iron–sulfur clusters, which are integral components of hydrogenases. To address this challenge, we employ the Extended Broken-Symmetry method for the computation of the cubane–[Fe_3_S_4_] cluster within the [FeNi] hydrogenase enzyme. This approach rectifies the error caused by spin contamination, allowing us to obtain the magnetic exchange coupling constant and the energy level of the low-lying state. We find that the Extended Broken-Symmetry method provides more accurate results for differences in bond length and the magnetic coupling constant. This accuracy assists in reconstructing the low-spin ground state force and determining the geometric structure of the ground state. By utilizing the Extended Broken-Symmetry method, we further highlight the significance of the geometric arrangement of metal centers in the cluster’s properties and gain deeper insights into the magnetic properties of transition metal iron–sulfur clusters at the reaction centers of hydrogenases. This research illuminates the untapped potential of hydrogenases and their promising role in the future of photosynthesis and sustainable energy production.

## 1. Introduction

In the face of the energy crisis and the imperative of climate change mitigation, societal growth and development have increasingly depended on fossil energy. However, as we continue to exploit these resources, their depletion is becoming inevitable. Moreover, the use of fossil fuels results in substantial greenhouse gas emissions, which contribute to the greenhouse effect and global warming [1,2]. Consequently, in order to align with the principles of green chemistry and clean technology, it is essential to intensify research and development in renewable energy, like hydrogen. Today, more than 95% of hydrogen is produced from hydrocarbons through steam reforming or partial oxidation. These methods are energy-consuming, and they are still dependent on fossil fuels, generating CO_2_, black carbon particles, and climate-relevant reactive gases as by-products. The establishment of a hydrogen-based economy remains a challenging task, and there is much one can learn from nature. Nowadays, there are several pathways to solve this problem by using different ways to produce and apply hydrogen, like making electrolytic devices [3], chemical fuel cells [4], artificial hydrogenases [5], and nanomaterials made of transition metal oxides [6]. The application of solar energy and hydrogenases is also a possible way, which is called photosynthesis. At present, photosynthesis is the only process that can gently split water into electrons and hydrogen [7,8]. However, the conversion efficiency of natural photosynthetic systems remains low [9,10,11]. Therefore, enhancing this conversion efficiency through artificial means is of significant importance.

In the photosynthetic system, the catalytic conversion reaction is primarily facilitated by hydrogenase [12]. Hydrogenase is the enzyme catalyzing the interconversion of hydrogen into protons and electrons (hydrogen ↔2H^+^+2e^−^) in bacteria, archaea, and eukaryotes [13]. Even though several microorganisms using hydrogen as an energy source attracted the attention of scientists in the 1800s, Stephenson and Stickland [14] were the first to propose the existence of hydrogenases and report the kinetic properties, as well as the oxygen sensitivity, of these enzymes. More recently, several crystal structures of hydrogenase have helped to unveil the geometry and mode of action of their active site [15], and extensive phylogenetic analyses have revealed that microorganisms harboring genes encoding hydrogenases encompass the three domains of the tree of life and are ubiquitous in the environment [16]. These enzymes are utilized to generate energy, disperse reducing equivalents produced during fermentation, or generate reduced cofactors involved in several reactions of cellular metabolism. In general, microorganisms utilize hydrogen under a mixotrophic lifestyle, which confers the ability to proliferate and survive in environments lacking readily available organic substrates. From an ecological perspective, hydrogen is viewed as a universal energy source, supporting a seed bank of hydrogen-oxidizing microorganisms that provide a broad range of ecosystem services [17]. Advances in our understanding of the biochemistry, diversity, and functions of hydrogenases contribute to the development of new biotechnologies and a better understanding of the hydrogen cycle and the ecological role of hydrogen-oxidizing microorganisms.

The catalytic conversion carried out by hydrogenase predominantly occurs in transition metal clusters at its core. These clusters can serve as catalysts, continually providing protons or hydrogen [18]. A comprehensive understanding of these transition metal clusters within hydrogenases will aid in elucidating their catalytic processes, and this is crucial for the design of transition metal complexes that serve as potentially sustainable proton reduction or H_2_ oxidation catalysts. Hydrogenases can be classified into several types based on the differences in the central transition metal cluster, including [NiFe] hydrogenases, [FeFe] hydrogenases, and [Fe] hydrogenases [19]. Among these, [NiFe] hydrogenases represent a crucial category and can be further divided into two types: oxygen-tolerant [NiFe] hydrogenases and oxygen-sensitive [NiFe] hydrogenases [20]. However, oxygen-sensitive [NiFe] hydrogenases become inactive when exposed to oxygen, thus limiting their practical applications [21]. Consequently, the development of oxygen-tolerant [NiFe] hydrogenases presents a significant area of research.

**Figure 1:**
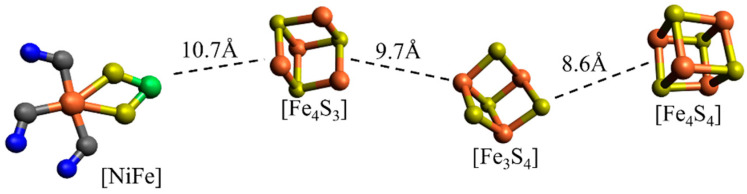
The diagram illustrates the [NiFe], [Fe_4_S_3_], [Fe_3_S_4_], and [Fe_4_S_4_] clusters involved in the catalytic pathway, as well as the distances between each cluster.

Within the protein fragment of oxygen-tolerant [NiFe] hydrogenases, there exists a catalytic conversion pathway in which the [NiFe] cluster serves as the central active site. The active site of the [NiFe] hydrogenases features a nickel tetrathiolate (four cysteines) with two S bridges to an Fe(CN)_2_(CO) center [22]. Four key states in the catalytic cycle are Ni-SI_a_ (Ni^II^Fe^II^), Ni–L (Ni^I^Fe^II^), Ni–C (Ni^III^µ(H)Fe^II^), and Ni–R (Ni^II^µ(H)Fe^II^) [23]. During the catalytic process, in the process of valence changes of iron and nickel, three electrons are required, which are supplied by [Fe_4_S_3_], [Fe_3_S_4_], and [Fe_4_S_4_] [24]. The relative positions and distances between these clusters are depicted in Figure 1. Existing studies indicate that the [Fe_3_S_4_] cluster plays a pivotal role in oxygen-tolerant [NiFe] hydrogenases [25,26]. Therefore, research focusing on [Fe_3_S_4_] clusters could represent a significant breakthrough in the development of oxygen-tolerant hydrogenases.

Currently, calculations of transition metal clusters, such as [Fe_3_S_4_] clusters, primarily rely on density functional theory (DFT) [27,28]. The Broken-Symmetry (BS) method is a common approach within DFT. This method calculates the energy difference between the high-spin state and the BS state, allowing for an estimation of the magnetic coupling constant, *J*, between spin centers [29]. However, because the BS state is not an eigenstate of the total spin operator *Ŝ*^2^ [30], some errors may occur when using the BS method to calculate the magnetic coupling constant *J.* Additionally, due to the strong magnetism of transition metal clusters and the high degree of dynamic and static correlation among the 3d orbital electrons of the central atom [31], clusters exhibit multiple degenerate states, with the ground state (GS) typically being a low-spin state. Concerning the static correlation, we may distinguish two different effects: the local static correlation necessary to correctly describe the nature of the bonds between each metal atom and its ligands and the global static correlation among the spin centers due to the interactions between localized unpaired electrons. This latter contribution is crucial to guaranteeing the correct overall spin symmetry of the wave function [32]. In the DFT method, the open-shell single-determinant wavefunction utilized in the Kohn–Sham equation fails to deliver the correct spin symmetry [33]; therefore, the DFT method cannot correctly describe the low-spin GS of the cluster, nor can it correctly calculate the cluster’s magnetic properties.

Previous studies have conducted a series of computational analyses on transition metal clusters, specifically those involving iron (Fe), manganese (Mn), and cobalt (Co) complexes within photosynthetic systems [34,35,36,37]. However, due to the limitations of the DFT method previously discussed, the calculated values of system energy and magnetic coupling constants *J* for multicenter transition metal clusters significantly deviate from experimental data. This discrepancy underscores the need to refine our computational methods to more accurately characterize the properties of transition metal clusters.

To rectify the errors resulting from the issues mentioned above, in this study, we will apply the Extended Broken-Symmetry (EBS) method [38] to perform calculations on the cubane–[Fe_3_S_4_] cluster. Through the Heisenberg–Dirac–van Vleck (HDvV) Hamiltonian, we aim to derive a low-spin ground state (GS) with correct symmetry for a cluster with an arbitrary number of spin centers. Based on preliminary calculations [39], for the low-spin GS, we will carry out multiple iterative optimizations on the geometric structure until the program converges. We will then obtain the magnetic exchange coupling constant *J*, energy levels, and energy spectral distribution of the final cluster structure. By comparing the EBS method with the BS method and the high-spin (HS) method, we find that the EBS method yields a bond length and magnetic coupling constant data that are closer to the experimental data, indicating a better description of the system. We anticipate that the EBS method will yield more accurate properties and structures of the low-lying state, which is closest to the eigenstate of transition metal clusters. As these clusters are the core catalytic oxidation reaction centers of hydrogenases, and the function of hydrogenase is carried out by the redox process of these clusters, obtaining more precise information on their magnetic properties and structure can lead to a deeper understanding of the nature of the hydrogenases in which they are incorporated.

## 2. Results and Discussion

### 2.1. Structure and Spin State

The object of our calculation is the [[Fe_3_S_4_](CH_3_CH_2_S)_3_(CH_3_CH_2_SH)]^3-^ cluster, with its central cluster being [Fe_3_S_4_]^1+^. In this cluster, all three Fe centers are Fe(III), and the 3d orbital contains five electrons.

**Figure 2:**
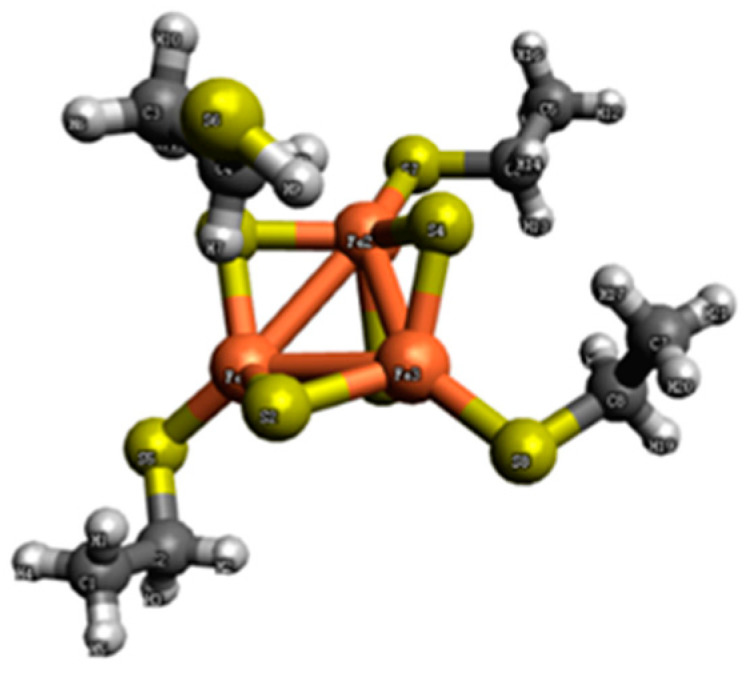
The structure of the [Fe_3_S_4_(CH_3_CH_2_SH)_3_]^2−^ complex. Iron is represented in orange, sulfur in yellow, carbon in black, and hydrogen in gray.

Initially, we characterized the clusters using the BS method. The central [Fe_3_S_4_]^1+^ cluster contains three Fe spin centers and four different BS states. We assume that the outermost electrons of each Fe atom have spins in the same direction at each spin center, implying that the spin at each spin center is *s* = 5/2; Therefore, the spins of the four different BS states are presented in Table 1. The clusters we selected were from existing research [40], and the cluster model is illustrated in Figure 2.

### 2.2. Bond Lengths between Spin Centers

We employed three methods—the HS method, the BS method, and the EBS method—to calculate the structure of the cluster and compared the results. The HS method calculates the geometry of the high-spin state of the cluster. The BS method calculates the four different BS states of the cluster. The details of EBS method can be found in Section 3.

The bond lengths of the cluster’s Fe–Fe bonds obtained from these calculations are presented in Table 2, where the unit of the bond length is Å.

From the table, it is evident that the B3LYP functional calculations using the HS method generally have a large deviation from the experimental data of approximately 0.3~0.4 Å, with an error of around 15%. The bond length error is about 0.2~0.3 Å, and the error percentage is roughly 10%. The discrepancy between the bond length data calculated using the EBS method and the experimental structure is approximately 0.1 Å, and the deviation percentages from the experimental data are 5% and 4%, respectively.

Similar conclusions were reached in the calculations using the TPSSh functional on clusters. We observed that the bond lengths calculated using the HS method have large discrepancies from the experimental values, resulting in a loose cluster structure. The cluster structure has been optimized to some extent using the BS method, but there is still a significant error in some bond lengths. However, the EBS structure optimization yielded a structure closest to the experimental data. Therefore, we believe that the results obtained using the EBS method are more accurate than those obtained using the HS and BS methods. Based on the above results, the HS, BS, and EBS methods, which give the smallest deviation, underestimate the bond length between spin centers. Bond lengths from the X-ray diffraction method were obtained for the solid phase, where the structure is distorted by intermolecular interactions. Meanwhile, the bond lengths from the HS, BS, and EBS methods were obtained for free molecular systems. Therefore, these methods fail to consider that the field generated by the external ligands and external molecules could be the reason for this underestimation.

**Figure 3:**
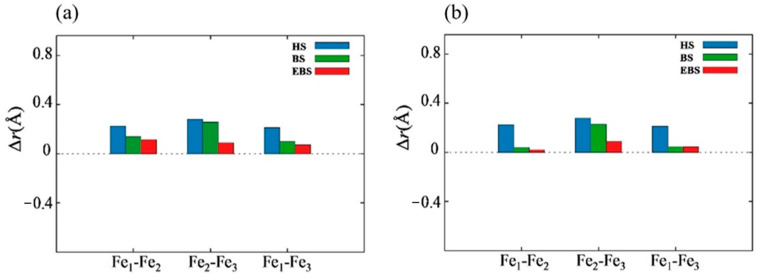
(**a**) Shows the comparison of Δ*r* using HS, BS, and EBS methods with the B3LYP hybrid functional. (**b**) Shows the same comparison using the TPSSh hybrid functional.

Figure 3a,b illustrate the comparison of the bond length difference, Δ*r*, which represents the differences between the experimental value and calculated value using the HS, BS, and EBS methods with the B3LYP and TPSSh functionals, respectively.

### 2.3. Exchange Coupling Constants

The magnetic coupling constants *J* calculated using the BS method and the EBS method are shown in Table 3, where *J*_1_ represents the magnetic coupling between Fe_1_ and Fe_2_, *J*_2_ represents the magnetic coupling between Fe_2_ and Fe_3_, and *J*_3_ represents the magnetic coupling between Fe_1_ and Fe_3_. In the EBS method, each structure optimization provides a new optimized structure and outputs its corresponding magnetic coupling constant *J*. For the optimized geometry obtained using the B3LYP functional, the corresponding *J* values are −109.5 cm^−1^, −119.8 cm^−1^, and −100.9 cm^−1^; For the converged geometry obtained using the TPSSh functional, the *J* values were −155.7 cm^−1^, −149.6 cm^−1^, and −124.3 cm^−1^, respectively.

In contrast with the range provided by experimental values, we observe that the magnetic coupling constants (*J* values) obtained using both the B3LYP and TPSSh functionals are smaller. From the Fe–Fe bond lengths in Table 2, it is suggested that as the Fe–Fe distance increases, the antiferromagnetic coupling becomes weaker. Therefore, we believe that the obtained *J* coupling constant aligns with the bond length data.

The *J* values calculated using the BS and EBS methods revealed that the *J* coupling constants given by the BS method generally deviate significantly from the experimental values. After optimization using the EBS method, the *J* value is noticeably closer to the range provided by experimental values, reducing the error by approximately 15% compared to the BS method with the same functional. Furthermore, a comparison of the *J* coupling constants and bond lengths indicates that the EBS-optimized structure is denser and has shorter bond lengths than the one optimized using the BS method, leading to stronger magnetic coupling. In summary, we believe that the EBS method reduces the calculation error to a certain extent, and the structure obtained using the BS method, once optimized through the EBS method, yields a compact cluster structure closer to the experimental data. Based on the calculations we have performed, we believe that the best results can be obtained by using the EBS method and the TPSSh hybrid function.

Compared to the linear–[Fe_3_S_4_] [38] cluster, which has two similar exchange coupling constants, the cubane–[Fe_3_S_4_] cluster in this study provides three similar exchange coupling constants, as shown in Table 3. This could generate more nearly degenerate states and enrich the properties of the cluster as a catalyst. The spatial configuration of the transition metal atoms in a cluster plays an important role in determining its electron conductivity and magnetic properties.

### 2.4. Energy Spectrum

**Figure 4:**
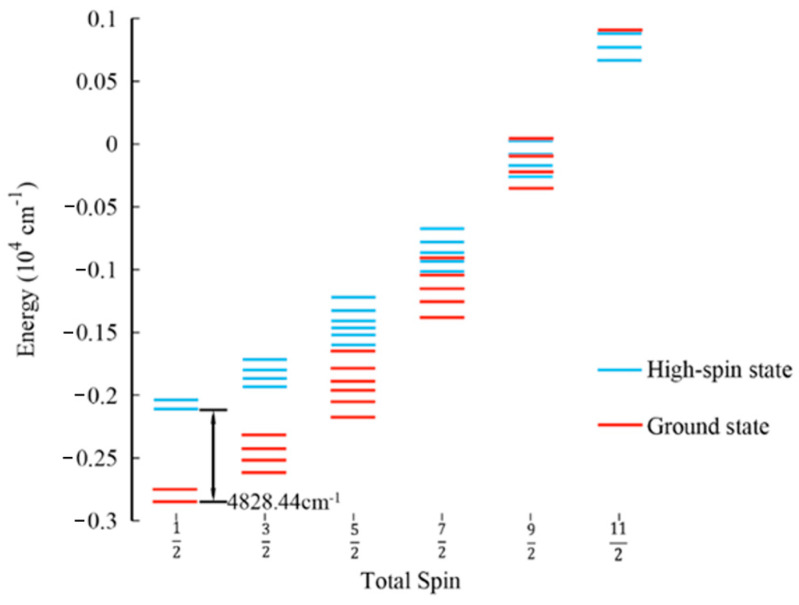
Comparison of the spin ladder of the GS and high−spin state calculated using the B3LYP functional. The blue lines represent the spin ladder of the high−spin state, and the red lines represent the spin ladder of the GS. After the optimization of the structure using the EBS method, the calculated energy difference between GS and HS states is 4828.44 cm^−1^.

The energy spectral distributions of the HS state and the GS state after EBS optimization using the B3LYP functional are depicted in Figure 4. The energy spectral distribution reveals multiple degeneracies in the low-spin GS of the [[Fe_3_S_4_](CH_3_CH_2_S)_3_(CH_3_CH_2_SH)]^2−^ cluster. The small gap between energy levels suggests that the cluster is susceptible to redox reactions when perturbed. Additionally, upon comparing the cluster energies calculated using the two methods, the cluster energy calculated using the HS method is −7294.034 Eh, while the cluster energy calculated using the EBS method is −7294.056 Eh. We found that structure optimization through EBS can achieve a lower energy, with an energy difference of 0.022 Eh (4828.44 cm^−1^).

**Figure 5:**
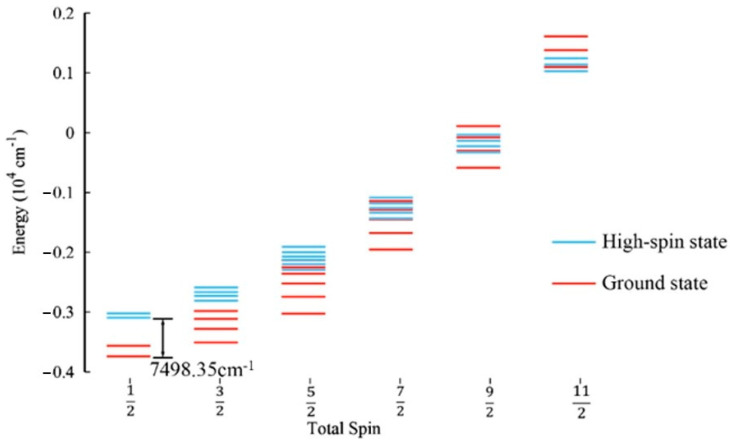
Comparison of the spin ladder of the GS and high–spin state calculated using the TPSSh functional. The blue lines represent the spin ladder of the high–spin state, and the red lines represent the spin ladder of the GS. After the optimization of the structure with the EBS method, the calculated energy difference between GS and HS states is 7498.35 cm^−1^.

The energy spectral distributions of the HS state and the GS state after EBS optimization using the TPSSh functional are depicted in Figure 5. The cluster energy calculated using the HS method is −7295.034 Eh, and the cluster energy calculated using the EBS method is −7295.144 Eh. We find that the structure optimization of the cluster using the EBS method can achieve a lower energy. The energy difference between them is 0.034 Eh (7498.35 cm^−1^).

## 3. Computational Method

In this calculation, ORCA [43] software was used. The all-electronic Ahlrichs TZVP [44] and Def2-TZVP [45] basis sets were chosen. The self-consistent field (SCF) convergence criterion was set to TightSCF and Grid4. The B3LYP and TPSSh hybrid functionals were chosen for this study. In addition to the B3LYP hybrid function, which is most commonly used in the calculation of transition metal clusters, we also selected the TPSSh hybrid function benchmarked on transition metal diatomics. This choice was motivated by findings that the TPSSh functional produces structures of comparable quality to those obtained using other commonly used hybrid and non-hybrid functionals, such as B3LYP and BP86. Moreover, the inclusion of 10% exact exchange in TPSSh can eliminate the large systematic component of the error, providing an advantage over other functionals [46].

In this study, we used the Extended Broken-Symmetry (EBS) calculation method to compute the properties of clusters. The detailed derivation has been described in a previous study [38]. Our primary results include the magnetic coupling constant *J* and the optimized energy spectral distribution of the cluster structure. The calculation process consists of several main steps.

In the first step, we calculate the energy *ε*^BS^ of each BS state using the BS method. Furthermore, we apply the EBS method using the results we obtained from the BS method, as follows. We construct the matrix ***A****_kp_* = $\langle s_{i}\cdot s_{j}\rangle$, and a linear equations system is defined in Equation (1):

(1) $$\left(\begin{array}{c} {\varepsilon_{1}}^{\mathrm{BS}}\\ {\varepsilon_{2}}^{\mathrm{BS}}\\ {\varepsilon_{3}}^{\mathrm{BS}}\\ \vdots \\ {\varepsilon_{N_{k}}}^{\mathrm{BS}} \end{array}\right)=-2\left(\begin{array}{cccc} A_{11} & A_{12} & \cdots & A_{1N_{p}}\\ A_{21} & A_{21} & \cdots & A_{2N_{p}}\\ A_{31} & A_{31} & \cdots & A_{3N_{p}}\\ \vdots & \vdots & \ddots & \vdots \\ A_{N_{k}1} & A_{N_{k}2} & \cdots & A_{N_{k}N_{p}} \end{array}\right)\left(\begin{array}{c} J_{1}\\ J_{2}\\ J_{3}\\ \vdots \\ J_{N_{p}} \end{array}\right)$$

After rewriting the above equations as matrix ***A***, we obtain the inverse matrix ***A***^−1^ of the matrix ***A****_kp_* via singular value decomposition (SVD) [39]. The magnetic coupling constant ***J*** is then obtained from Equation (2).

(2) $$J=-\frac{1}{2}A^{-1}\cdot \varepsilon^{BS}$$

Secondly, the Hamiltonian matrices ⟨*b*_l_| and |*b*_r_⟩ are constructed, where ⟨*b*_l_| represents the left basis vector of each eigenstate and |*b*_r_⟩ represents the right basis vector of each eigenstate. Using the ***J*** coupling constant obtained from Equation (2) and the Clebsch−Gordan (CG) coefficient [47], we can diagonalize the Hamiltonian, as indicated in Equation (3). The CG coefficient essentially functions as a transformation matrix for representations grounded in group theory, and it is capable of converting an uncoupled representation into a coupled one [48]. In the case of the cubane–[Fe_3_S_4_] cluster, we can describe the low-spin GS through the combination of the CG coefficients and each BS state.

(3) $$\begin{array}{cc} \langle b_{l}\left|\hat{H}\right|b_{r}\rangle & =-2\sum_{i<j}\langle b_{l}\left|J_{ij}{\hat{s}}_{i}\cdot {\hat{s}}_{j}\right|b_{r}\rangle \\ & =-\sum_{i<j}J_{ij}\langle b_{l}\left|{{\hat{S}}_{ij}}^{2}\right|b_{r}\rangle +\sum_{i<j}J_{ij}\langle b_{l}\left|{{\hat{s}}_{i}}^{2}\cdot {{\hat{s}}_{j}}^{2}\right|b_{r}\rangle \\ & =-\sum_{i<j}J_{ij}\times CG_{ij}^{l}\times CG_{ij}^{r}\times S_{ij}\left(S_{ij}+1\right)\\ & +\sum_{i<j}J_{ij}\times \left(s_{i}\left(s_{i}+1\right)+s_{j}\left(s_{j}+1\right)\right) \end{array}$$

**Figure 6:**
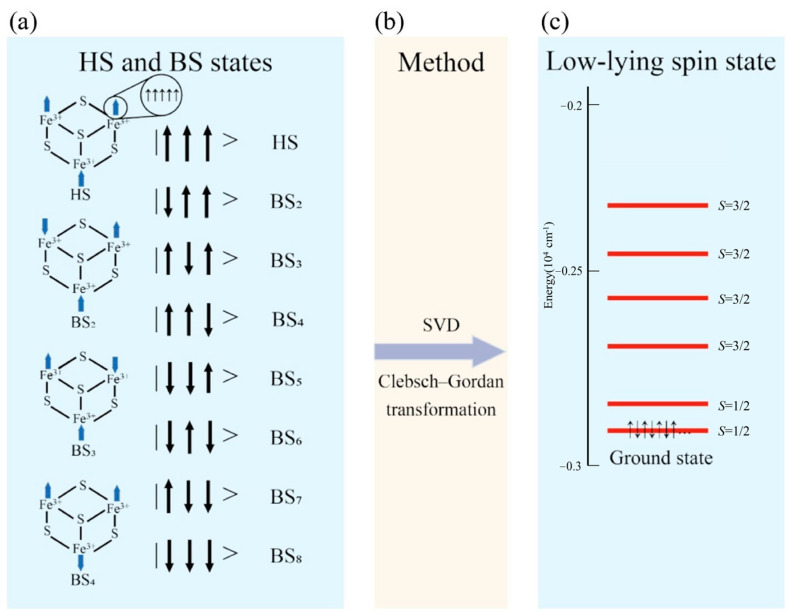
(**a**) Depicts the 8 broken symmetry states of the cubane–[Fe_3_S_4_] cluster, complete with spin details. The energy level of the low-lying state (LS state) is illustrated in (**c**), where the total spins *S*_tot_ of 1/2 and 3/2 are shown. This implies that there are 7 pairs of electrons and 1 single electron in the state with *S* = 1/2 and 6 pairs of electrons and 3 spin-up electrons in the state with *S* = 3/2. The energy in (**c**) is derived from calculations of cubane–[Fe_3_S_4_] clusters using the B3LYP functional. The magnetic coupling constant *J* can be extracted from the SVD matrix. The Clebsch–Gordan transformation, represented in (**b**), enables the description of the cluster’s GS.

Through the above two steps, we can obtain the GS structure and its corresponding energy spectral distribution of the cluster, as shown in Figure 6.

**Figure 7:**
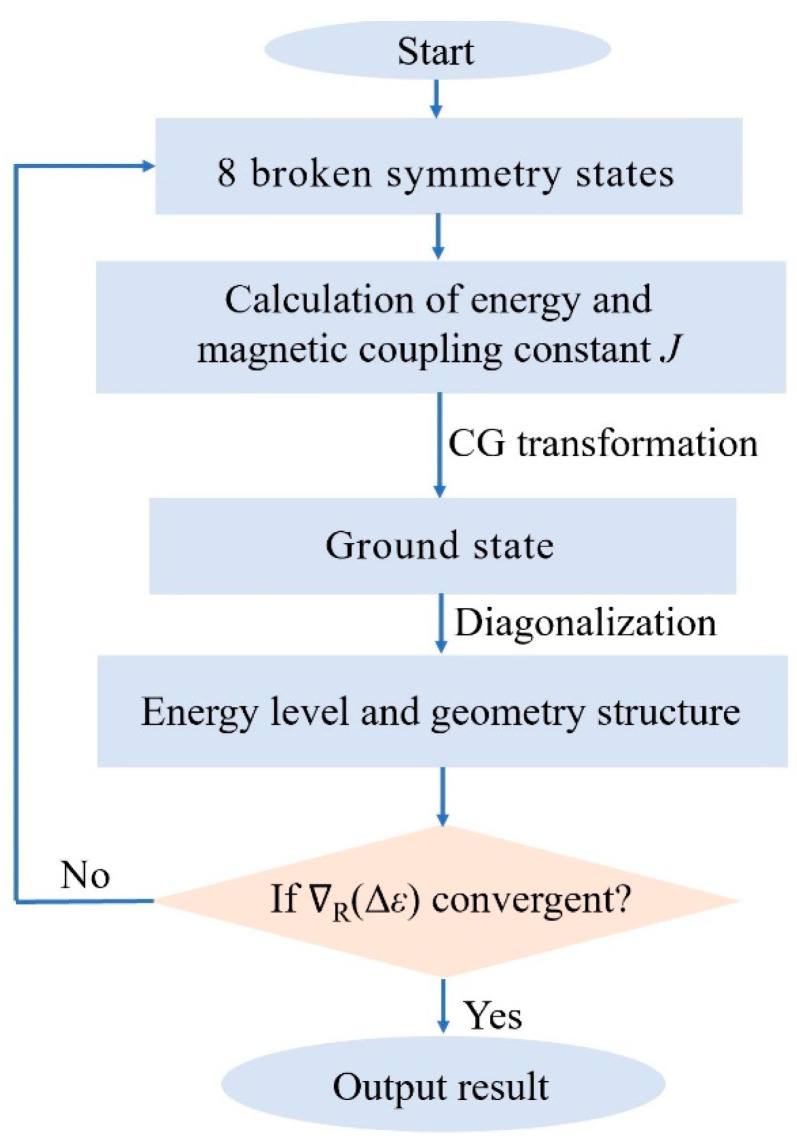
Flow chart of the geometric optimization of clusters using the EBS method.

Having completed all of the calculations above, we have obtained the energy of all BS states, the magnetic coupling constant *J*, and the optimized structure, along with its GS. Once we have these preliminary results, we can perform geometry optimization using the EBS method, a FORTRAN code generated by interfacing the external optimizer features available in ORCA. Figure 7 provides a schematic diagram of the entire calculation process.

We investigated the energy gradient, denoted as ∇_R_(Δ*ε*), with further details provided in the previous study [38].

When ∇_R_(Δ*ε*) becomes less than the convergence value, it indicates that the optimized GS geometry has achieved our required accuracy. At this point, the program terminates, and the final GS geometry, magnetic coupling constant *J*, and energy spectral distribution are output.

## 4. Conclusions

We calculated the energies of all BS states of the cubane–[Fe_3_S_4_] cluster via the DFT method and obtained the GS energy spectrum structure via the SVD method and CG transformation. Based on this ground-state energy surface, we further optimized the geometry and energy spectral distribution of the low-spin GS. We find that compared to those obtained through the BS method, the geometric parameters calculated using the EBS method can match better with the experimental data. Therefore, we believe that the EBS method compensates for the shortcomings of the BS method used in the DFT method and that it reduces the errors caused by the static correlation and spin contamination. From the energy spectrum, it is evident that the [Fe_3_S_4_] cluster possesses rich magnetic properties, suggesting that [Fe_3_S_4_] clusters could serve as exceptional mediators of electron conductivity. Furthermore, the nearly equal *J* values of the three magnetic coupling constants in [Fe_3_S_4_] clusters could be a crucial factor contributing to the robust oxygen tolerance of [NiFe] hydrogenase. These *J* values are determined according to the spatial configuration of the transition metal atoms within the cluster, further highlighting the significance of the metal center’s geometric arrangement in the cluster’s properties. The EBS method represents an important step toward the precise study of transition metal clusters. We believe that for multiple magnetic clusters, it is necessary to consider the static correlation effect and perform quantitative comparisons with the experimental data to deepen the understanding of the clusters. We hope that the study of magnetic properties and energy spectral distributions can help us better understand transition metal clusters and their functions and properties in hydrogenases.

## Figures and Tables

**Table 1:** Different spin states of the [Fe_3_S_4_]^1+^ cluster.

*BS_k_*	Spin State	$|s_{1},s_{2},s_{3}\rangle$	*S*_tot_
*BS*_1_	↑↑↑	$|\;\;\frac{5}{2},\frac{5}{2},\frac{5}{2}\rangle$	$\frac{15}{2}$
*BS*_2_	↓↑↑	$|-\frac{5}{2},\frac{5}{2},\frac{5}{2}\rangle$	$\frac{5}{2}$
*BS*_3_	↑↓↑	$|\frac{5}{2},-\frac{5}{2},\frac{5}{2}\rangle$	$\frac{5}{2}$
*BS*_4_	↑↑↓	$|\frac{5}{2},\frac{5}{2},-\frac{5}{2}\rangle$	$\frac{5}{2}$

**Table 2:** Bond length of the [[Fe_3_S_4_](CH_3_CH_2_S)_3_(CH_3_CH_2_SH)]^2−^ cluster. The experimental data are from the X-ray structure analysis [41]. Errors are calculated according to the differences between the experimental value and the calculated value.

Hybrid Function	Method	Fe_1_-Fe_2_/Å	Fe_2_-Fe_3_/Å	Fe_1_-Fe_3_/Å	Error/%
**Exp [41]**		2.71	2.67	2.73	
B3LYP	HS	3.05	3.07	3.06	12%~15%
	BS	2.88	3.03	2.90	6%~13%
	EBS	2.85	2.79	2.87	4%~5%
TPSSh	HS	2.99	3.02	2.99	10%~13%
	BS	2.76	2.95	2.79	2%~8%
	EBS	2.73	2.78	2.78	0.7%~4%

**Table 3:** Magnetic spin coupling *J*/cm^−1^ between spin centers calculated using the BS and EBS methods with B3LYP and TPSSh hybrid functionals. The experimental data are from the X-ray structure analysis [42].

Hybrid Function	Method	*J*_1_/cm^−1^	*J*_2_/cm^−1^	*J*_3_/cm^−1^
B3LYP	BS	−65.4	−67.5	−62.1
	EBS	−109.5	−119.8	−100.9
TPSSh	BS	−94.0	−89.0	−88.5
	EBS	−155.7	−149.6	−124.3
Exp [42]		−(200~300), *J*_1_ ≈ *J*_2_ ≈ *J*_3_		

## Data Availability

Data are contained within the article.
